# Household electricity consumption prediction using database combinations, ensemble and hybrid modeling techniques

**DOI:** 10.1038/s41598-024-57550-9

**Published:** 2024-10-02

**Authors:** Gaikwad Sachin Ramnath, R. Harikrishnan, S. M. Muyeen, Ketan Kotecha

**Affiliations:** 1 Symbiosis Institute of Technology (SIT), Pune Campus, Symbiosis International (Deemed) University, Pune, India; 2 Department of Electrical Engineering, Qatar University, 2713 Doha, Qatar; 3 Symbiosis Centre for Applied Artificial Intelligence, Symbiosis Institute of Technology, Symbiosis International (Deemed) University, Pune, India

**Keywords:** Household electricity consumption, Data quality assessment, Monthly prediction, Heterogeneous ensemble, Hybrid model, Energy science and technology, Engineering

## Abstract

Household electricity consumption (HEC) is changing over time, depends on multiple factors, and leads to effects on the prediction accuracy of the model. The objective of this work is to propose a novel methodology for improving HEC prediction accuracy. This study uses two original datasets, namely questionnaire survey (QS) and monthly consumption (MC), which contain data from 225 consumers from Maharashtra, India. The original datasets are combined to create three additional datasets, namely QS + MC, QS equation (QsEq) + next month’s consumptions, and QsEq + MC. Furthermore, the HEC prediction accuracy is boosted by applying different approaches, like correlation methods, feature engineering techniques, data quality assessment, heterogeneous ensemble prediction (HEP), and the hybrid model. Five HEP models are created using dataset combinations and machine learning algorithms. Based on the MC dataset, the random forest provides the best prediction of RMSE (36.18 kWh), MAE (25.73 kWh), and R^2^ (0.76). Similarly, QsEq + MC dataset adaptive boosting provides a better prediction of RMSE (36.77 kWh), MAE (26.18 kWh), and R^2^ (0.76). This prediction accuracy is further increased using the proposed hybrid model to RMSE (22.02 kWh), MAE (13.04 kWh), and R^2^ (0.92). This research work benefits researchers, policymakers, and utility companies in obtaining accurate prediction models and understanding HEC.

## Introduction

The world’s residential energy demand is increasing due to continuously rising population size, and socio-economic and technological development^1^. Utility companies are challenged to reduce the demand–supply gap without affecting human and environmental development^2^. Besides, integrating renewable and non-renewable energy sources can empower electric grids. There is a challenge to incorporating both sources, which is the occurrence of interference and volatility issues. In this regard, a soft computing technique can solve the problems. Accurate energy prediction of household electricity consumption (HEC) is essential to obtain effective energy management, energy conservation, energy optimization, reducing excess electricity generation, reducing power outages, and detecting and diagnosing HEC faults^3–8^. In addition, energy consumption prediction models are essential for an informed decision, electricity markets, and various approaches have been developed as necessary^9^. Thus, accurate HEC prediction is an important research topic^10,11^. The better prediction accuracy of individual HEC is a challenging and complex task. This is due to the HEC being dependent on various indoor and outdoor parameters like socioeconomic status, demographics, the number of appliances and their usage, household characteristics, consumer lifestyle, weather conditioning, occupant energy behavior, etc.^12–18^. However, the energy consumption pattern or relationship within in dataset shows the linear, linear, non-linear, or nonstationary behaviour, which may also be affecting the prediction accuracy^3^.

In this aspect, Khan et al.^19^ discussed electric load prediction, types of predations, methods, advantages, and limitations. Similarly, a comparison of traditional and contemporary smart prediction approaches was conducted. The HEC prediction is broadly classified into three approaches: engineering, Artificial Intelligence (AI), and hybrid. Engineering and hybrid are the conventional energy prediction approaches that work on thermodynamic equations^8^. Due to the digitalization and smart meter connectivity, the historical dataset is readily available with a sufficient sample size. These historical datasets are required for developing an AI-based prediction model^9^. Compared to the engineering method, the advantage of the AI-based method is that it can quickly and effectively solve challenging problems that arise in real-time^20^.

Additionally, there are two ways the AI methodology can be implemented: single prediction methods and ensemble prediction methods. Compared to single prediction methods, ensemble methods have produced accurate prediction outcomes, according to the literature review. The ensemble model is advantageous in that it can accommodate both linear and non-linear datasets, as well as a wide range of input, and attributes that can be used, tackle complex energy consumption issues, and interact with predictors at multiple levels, with no assumption, and so on^5,21^. In addition, the ensemble machine learning (ML) method is mainly used to boost the prediction performance of single-prediction model results. The results of^10^ showed that the ensemble technique had improved HEC prediction model performance. This leads to support for the effective HEC management system. Due to this, AI-based ensemble methods are widely used for prediction model development^4,8^.

Tran et al.^1^ applied real and literature-based datasets to propose a new ensemble model for HEC prediction. The ensemble model used least squares support vector regression (LSSVR) and the radial basis function neural network (RBFNN) algorithms. In addition, the symbiotic organism search (SOS) approach is applied for auto-fine-tuning hyperparameters. The results analysis shows the proposed model performance concerning root mean square Error (RMSE) (36.31 kWh), mean absolute error (MAE) (29.45 kWh), mean absolute percentage error (MAPE) (8.90%), and coefficient of determination (R2) (0.93). This study identified two drawbacks. It requires more calculation time and cannot show a correlation between predictor and response qualities^1^.

Jovanović et al.^9^ implemented an energy consumption prediction approach for the university campus. In this, a feed-forward back propagation neural network (FFNN), radial basis function network (RBFN), and adaptive neuro-fuzzy interference system (ANFIS) were used. The real datasets of the coldest period of the year are used for developing the prediction model. Three neural network (NN) models are generated based on the different input parameters, and a comparative analysis of prediction results has been done. In addition, the ensemble method has provided a more accurate prediction performance than the particular method^9^.

Carmen Ruiz-Abell et al.^4^ presented bagging, random forest, conditional forest, and boosting methods to the electric load dataset of residential premises of the university campus. To improve the prediction accuracy, the authors have has applied temperature, calendar variables, and various special-day datasets. The random forest (RF) and gradient boosting (GB) methods have reduced around 11% electricity costs for university campus consumers^4^. The author^18^ has proposed a novel HEC prediction model using the ensemble method. The ensemble model combines GB regression, multi-layer neural networks, long-short-term memory (LSTM) networks, and linear repressor (LR). The ensemble model provided the best prediction results for MAPE (1.59%), RMSE (6.19 kWh), and MAE (5.60 kWh). The study commented that lower granularity in training datasets could reduce prediction accuracy^18^.

The individual HEC energy flexibility assessment faced difficulties due to the deficiency of a scalable, practicable approach. This problem has been discussed in^24^ by using ensemble methods with various algorithms, namely RF, multilayer perceptron neural network, support vector machine (SVM), and extreme GB. A study has applied dynamic attribute selection and HEC patterns to develop day-ahead and hour-ahead prediction models^24^. Fan et al.^22^ have developed an ensemble model to predict accurate day-ahead HEC, and the maximum power demand concerning MAPE is 2.32% and 2.85%, respectively. The author applied various techniques to improve prediction performance, outlier detection, feature engineering, selecting attributes, clustering analysis, and eight predictive models-based ensemble approach^21^. Jetcheva et al.^21^ proposed an NN-based ensemble method for day-ahead residential electric load and generation forecasting. In addition, authors have used the auto-selected hyperparameters approach for developing forecasting models^21^.

Burger and Moura^5^ implemented a generalized approach for predicting residential and commercial building energy consumption across geographic locations, seasons, and use types. For this, an ensemble method is used by incorporating an artificial neural network (ANN) or Seasonal Autoregressive Integrated Moving Average model. The prediction model has provided an absolute percent error (APE) performance of 7.5% and 55.8% for commercial and residential buildings, respectively^5^. Taylor and Buizza^23^ worked using the ensemble method of the ANNs model that applied electric load and weather datasets for one to ten days ahead of load prediction. The specialty of ANNs is to capture the unspecified nonlinear associations between electric load and weather conditioning variables^23^. Karijadi and Chou^3^ developed a hybrid prediction model using RF and LSTM algorithms. For this, two steps have been applied; in the first step, the original dataset has been decomposed.

The second step combines RF and LSTM to get the final best HEC prediction results. The limitation of this study is the only univariate (energy consumption dataset) prediction model with an hourly prediction scale has been used^3^. Khan et al.^19^ proposed a novel hybrid model for energy consumption prediction. The hybrid model combines three algorithms: multi-layer perceptron (MLP), SVR, and CatBoost. The prediction model used both renewable and non-renewable datasets of electric load and the time series’ characteristics datasets. At the same time, the study used various evaluation parameters, namely MAE, R^2^ mean squared error, and RMSE.

Increasing electricity demand is due to technological advancement and overall socio-economic development in society. Demand for electric vehicles (EVs) is increasing daily, and if we use non-renewable resources like coal, it causes environmental issues like global warming. This problem can be handled by promoting smart grid (SG) development by adding distributed energy sources through renewable resources and effective demand response management (DRM)^12–14^. For the SG and DRM, getting accurate load forecasting and prediction accuracy of energy consumption plays a significant role. In this scenario, the proposed work on developing an HEC precision modeling touches the needed broader literature^15,24^.

The organization of the paper includes four sections. Section "Proposed novel methodology for the MAP model" proposes a novel methodology to improve the accuracy of the MAP model using ML, ensemble, and hybrid approaches. Section "Results and discussion" discusses the results and their interpretation. Section "Conclusion and future scope" summarizes the outcome of the proposed novel methodology for the MAP model with future scopes.

### Novelty and contribution

**Figure 1:**
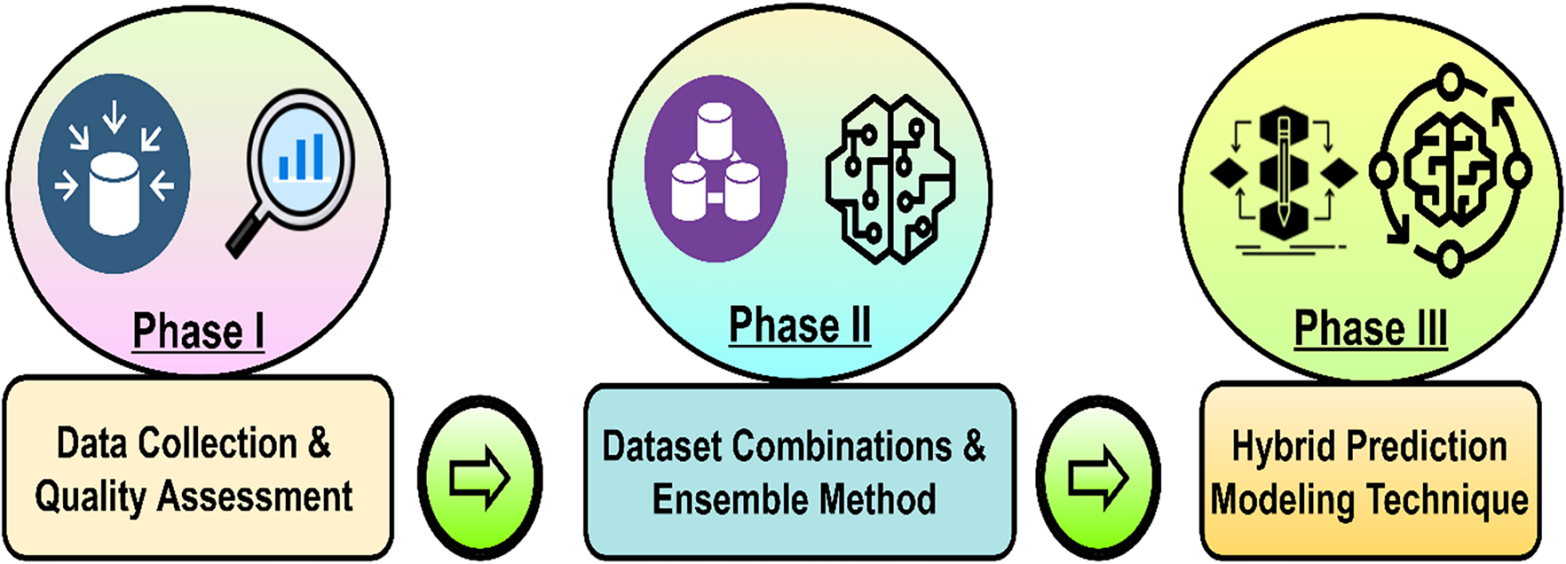
The generalized workflow of contributing to HEC prediction modeling.

There is a constraint to getting higher HEC prediction accuracy, which is due to its dynamic and multi-factors dependency^12^. This directly affects demand response management and other analyses^5,18,22^. This research aims to increase the HEC prediction accuracy of the month-ahead prediction (MAP) model through a novel methodology approach. The proposed work boost prediction accuracy by using a combination of algorithms, specific real datasets used, namely QS and MEC, features engineering based on newly generated features, and steps taken for data quality improvement. The generalized workflow for improving the prediction accuracy of HEC through a 3-phased approach, is shown in Fig. 1. These phases clearly indicate the process and major components involved in the prediction model.

In the first phase, the data Quality Assessment (QA) focused on data quality improvement. The second phase is to identify and select the influencing datasets and perfect fit algorithms. The third phase uses a hybrid prediction approach to increase the prediction accuracy of the MAP model^20^. The implementation and results analysis of the proposed work are discussed in Sections "Proposed novel methodology for the MAP model" and "Results and discussion".

Following are the objectives and contributions of this work.

1. A novel methodology for increasing HEC prediction accuracy has been proposed.
2. The finest dataset identification using the data QA approach is implemented.
3. A comparative study of five prediction models using a heterogeneous ensemble method is performed.
4. The hybrid model is proposed to boost the prediction accuracy of the MAP model.

## Proposed novel methodology for the MAP model

**Figure 2:**
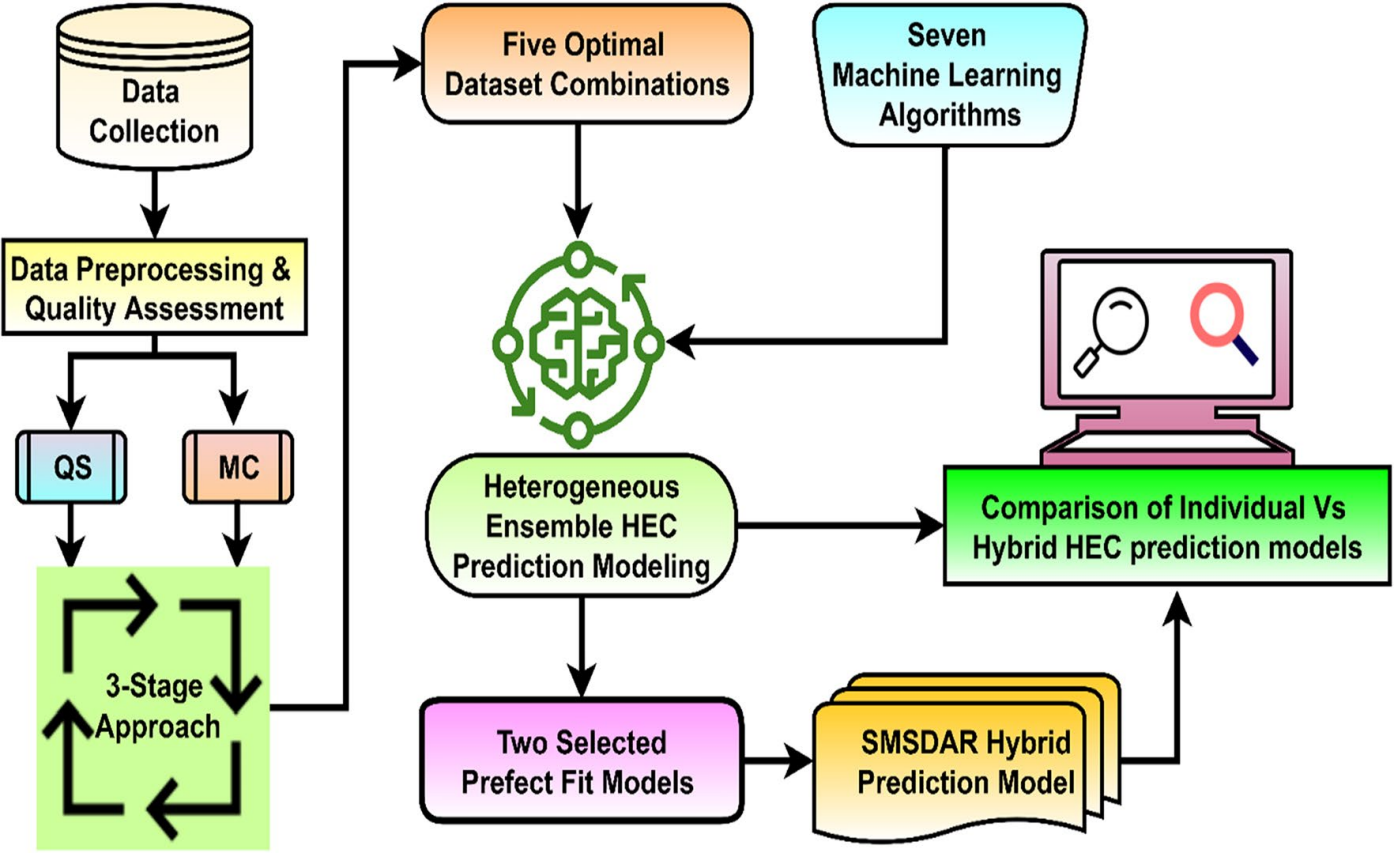
A workflow of the proposed methodology for HEC prediction modeling. *Note*: QS: Questionnaire survey; MC: Monthly consumption; HEC: Household electricity consumption; MLR: Multiple linear regression; SVM: Support vector machine; DT: Decision tree; RF: Random Forest; MLP: Multi-layer perceptron; AB: Adaptive boosting; SMSDAR: SVM + MLR + SGD + DT + AB + RF.

This innovative approach encompasses, namely, data collection and selection, data quality assessment, heterogeneous ensemble modeling with comparison, and a proposed hybrid prediction model. Figure 2 shows the workflow of the proposed work for the MAP model. This research introduces a novel methodology to enhance prediction accuracy, addressing the unique challenges of HEC data. The study precisely implements a data QA approach to identify the finest dataset, ensuring the reliability of input data. A comparative study of five prediction models which uses a Heterogeneous Ensemble Method has been done. Each model is briefly introduced along with the evaluation parameters, contributing to a robust evaluation process. Additionally, the research proposes a hybrid model designed to boost the prediction accuracy of the MAP model, integrating components strategically. Overall, these contributions advance HEC prediction capabilities, offering a multifaceted approach that encompasses methodological innovation, data quality assurance, comparative analysis, and a novel hybrid model. The subsequent sections discuss the implementation of the proposed methodology.

### Data collection, cleaning and data quality assessment approaches

**Figure 3:**
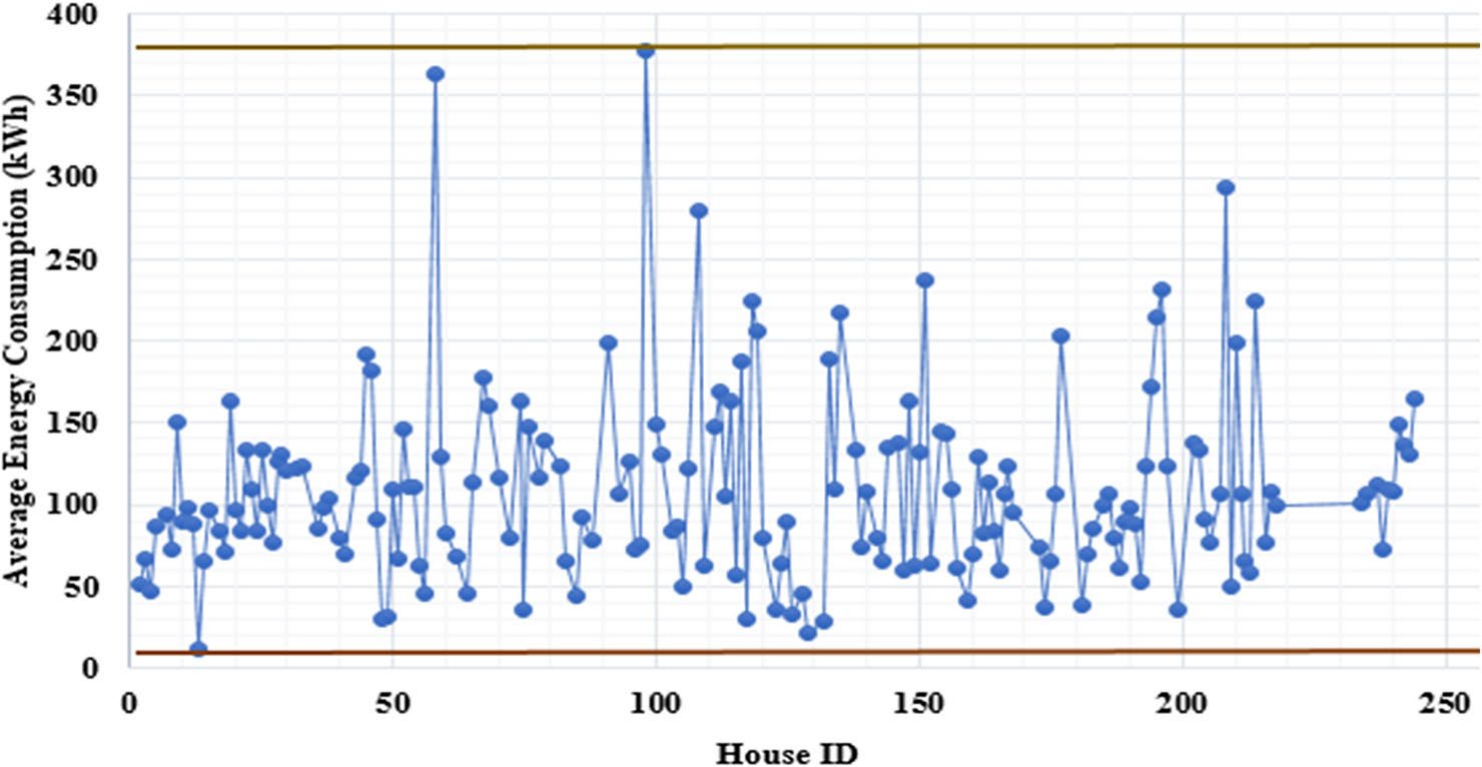
House’s monthly average energy consumption analysis by control chart.

The proposed work uses both primary and secondary data. The questionnaire survey (QS) method is used to collect household energy consumption-related data from different geographical locations, which is primary data. Around 225 household’s data was considered of Pune, Nashik, and Ahmednagar districts of Maharashtra, India^13^. The QS is designed and developed based on the problem statement. Further it is verified, corrected using domain knowledge and taking inputs from utility company engineers. The monthly energy consumption (MC) data is collected by QS and utility companies with primary and secondary data. The MC dataset includes 40 months of energy consumption dataset from January 2019 to April 2022. Figure 3 shows the analysis of the average energy consumption by houses. Around 73% of houses are within the average consumption range of 50–150 kWh. Meanwhile, only 7% of houses have an above-average consumption of 200 kWh.

The QS dataset comprises six parts: consumers’ basic information, monthly electricity bills, house characteristics, socio-demographic factors, appliance characteristics, feedback, and awareness information^12–17^. Besides, a detailed discussion on the QS data used is provided in^13^ with a list of factors and variables.

After data collection, the original or raw data is converted into understandable, improved quality with a required form of data. For this, data cleaning is a vital step before the data sets are applied to the prediction model. This work uses both primary and secondary data, which has been cleaned accordingly. As the data is a real word related to HEC, different scenarios are considered to maintain the data quality. To name a few, the COVID-19 pandemic situation, faulty energy meters, average bills are considered^13^. Even the MEC data is secondary but we cannot use in its original form due observed issues like discontinued or higher variations in monthly consumption and average monthly energy consumption in the history data. In addition, a normal or continuous and discontinues are the two energy consumption pattern are observed in the history household monthly energy consumption data. The discontinues consumption households are identified based on the lower monthly unit consumption between 0 and 3 kWh. This monthly low energy consumption can be due to house locked, house not in use due to on vacation, out of station, house temporary energy meter is disconnected etc.

There are two main household energy bills namely normal and average bill. A normal bill is generated based on the actual monthly energy consumption recorded in energy meter and average bill is calculated based on average of previous three months’ energy consumption. The average bill can be generated due to faulty energy meter, home locked, meter reading not taken, pandemic situation etc. Furthermore, the discontinued and average data is affecting on accuracy of prediction model and actual energy demand of individual household. This is because the MC dataset has the problem of discontinued energy consumption for 49 houses, and around 43 dwellings received average bills^13^. A total of 179 samples of residential household data after preprocessing is fed to the HEC prediction model development.

As per the proposed methodology and data type, several data cleaning techniques, including detection and handling of missing values and outliers, data integration, encoding categorical variables, handling inconsistent and irrelevant data, text cleaning, feature engineering, data validation, and data quality assessment are adopted^12,15^. Clustering-based methods like K-Means, Hierarchical, and self-organizing map (SOM) are used to identify the missing values and outliers in the dataset. The missing value imputation has been done by using the statistical method of mean, mode, and median based on the type of attributes. In the proposed work, QS data is considered to be independent or an input feature, and MC data is considered to be a dependent or target feature. Further, the data integration technique combines two different datasets through household identification (HID) attributes. The outlier samples are identified and resolved using domain knowledge, data visualization, and clustering methods. Data monitoring, review, data recollection, attributes central tendency, and frequently occurring techniques are used for correcting and imputing the data. Google form tool is mainly used for the data collection. While designing the Google form taken care of avoid wrong entry or garbage data by providing the thresholds based on type of question as check point. Data monitoring and review techniques are used to cross-validate the inconsistencies and suspicious pattern data^23^.

**Figure 4:**
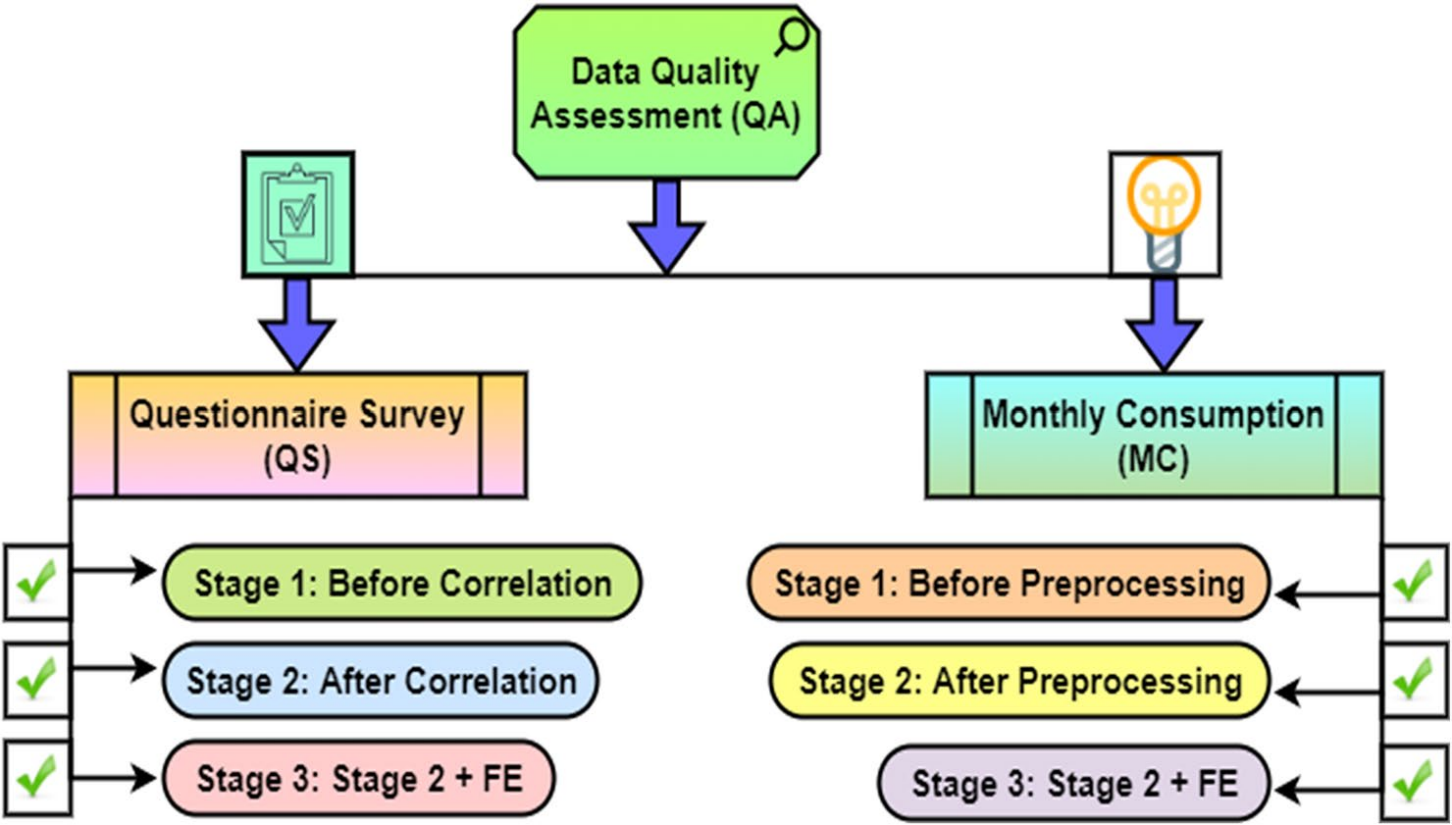
Data cleaning-based three-stage approach for data QA.

The selection of significant attributes, process simplicity, data interpretability, prevention of overfitting, boost resilience, and improved computational and data storage also plays a significant role in the prediction model performance. Based on the type of variables the Spearman and Pearson correlation method is used to identify the significant attributes and reduce the features. Feature engineering (FE) techniques are used to create a new significant attribute. Five features are created using the existing features of the QS dataset, namely the summation of socio-demographic factors, the total number of regular appliances, the total number of lifestyle appliances, the number of appliances older than five years, and the summation of significant features (QsSum). The QsSum feature is further used for household classification based on house size. Similarly, from MC dataset, five features are created, namely the average monthly energy consumption, maximum energy consumption, minimum energy consumption, standard deviation of MEC, and summation of monthly energy consumption. Overall the process of FE includes various steps like understanding the features, modifying features as per the application, and create feature as more informative and useful for the specific problem at hand. This approach comes under different techniques: feature extraction, feature transformation or scaling or normalization of data, feature selection, feature encoding and feature combination. A robust data QA approach to identify the finest dataset for the analysis is implemented. The process involves a three-stage approach for data QA as shown in Fig. 4. For the QS dataset, stages are before correlation, after correlation, and after correlation and FE. Similarly, MC dataset stages are before preprocessing, after preprocessing, and after preprocessing and FE. In addition, QS includes images and examples to understand questions easily. Moreover, expert knowledge-based data validation is also applied for data QA.

The MC dataset preprocessing stage is vital to remove irregular monthly consumptions and correct the monthly average consumptions. The attributes used and their impact on prediction accuracy are discussed in Section "Results and discussion", results and discussion. This QA approach ensures the selection of high-quality data, which is paramount for accurate predictions. This step-by-step procedure adds transparency to the dataset identification methodology.

### Machine learning algorithms for HEC prediction modeling

The AI-based ML algorithm prediction technique is based on the “black box” principle. The internal structure is unidentified in the black box mechanism; only the inputs and outputs are known^25^. The black-box approach is used because of not evaluate the underlying reasoning or decision-making process, the emphasis is on the model’s performance and its capacity to produce precise predictions^12–14^. Besides, the technique implemented was based on supervised ML due to label datasets. This proposed work uses ML algorithms namely Multiple Linear Regression (MLR), SVM, SGD, DT, RF, MLP, and AB for developing the heterogeneous ensemble and hybrid prediction model^14,15^. The selection of these algorithms and their HEC prediction involves considering the problem’s characteristics and each algorithm’s strengths. All these algorithms are selected to understand and enhance the HEC prediction performance based on their strengths and suitability for gathering various aspects of the data^1,3^. MLR provides simplicity, SVM and SGD handle complex relationships, DT and RF capture non-linear patterns, MLP handles NN capabilities, and AB addresses model weaknesses. Combining these algorithms can contribute to a comprehensive and accurate prediction model for HEC^12,22,23^. These algorithms were also selected for their efficacy in solving various real-world issues. Even though they may not be new, they are still frequently utilized, and they have gained popularity. It is important because of their simplicity, how effectively they can be utilized, and how effectively they can handle various data types. The aim of using ML algorithms to understand the problem and solve real-world problems is to boost prediction accuracy by proposing a heterogeneous ensemble prediction model and hybrid prediction model with novel methodology, unique features, and a real-world dataset^12,13^.

#### Multiple linear regression

Multiple Linear Regression (MLR) is a simple and interpretable algorithm that can provide linear relationships between input attributes and monthly energy consumption target attributes^12^. The outcome of MLR prediction can give clarity about the nature of the problem and the characteristics of the input and output attributes. In this problem, multiple input attributes of QS and MC datasets are available if there is a clear linear correlation between independent variables and electricity consumption. Then, prediction accuracy will be probably higher. Thus, MLR can provide a baseline model for HEC prediction, capturing straightforward relationships between various factors and household electricity consumption^19,22^.

In linear regression, the perfect fit linear line from the given datasets can be developed by assuming the association between input and target attributes. Based on the selection of several input variables, LR is of two types, namely simple linear regression (single independent variable) and MLR (more than two independent variables). This paper consists of one target attribute, month-ahead energy consumption in kWh, and multiple independent variables from QS and MC datasets. The mathematical representation of MLR is given by Eq. (1):

1 $$Y= {a}_{0}+{b}_{1}{X}_{1}+{b}_{2}{X}_{2}+\dots +{b}_{n}{X}_{n}+e$$

where $${X}_{1}$$ to $${X}_{n}$$, $$Y$$, and $$e$$ are the inputs, output, and error, respectively. The parameters, $${a}_{0}$$$${b}_{1}$$$${b}_{2}$$_,_ and $${b}_{n}$$ are the regression coefficients. In the case of real-time problems, sometimes MLR may face model overfitting or underfitting issues due to the non-linear dataset and complexity of the problem^12,25–27^. From this method, it was observed that the MLR model provides an overfitting problem. This problem has been tackled using appropriately fine-tuning hyperparameters. In contrast, MLR has limitations in dealing with non-linear datasets and high-dimensional attributes.

#### Support vector machine

Support Vector Machines (SVM) is operative in handling high-dimensional data and capturing non-linear associations between attributes. SVM is mainly suitable for both regression and classification problems. The SVM is beneficial because it can handle the complexity of HEC prediction, particularly when there are non-linear dependencies between the QS attributes and MC electricity consumption attributes^12,15,27^.

Authors Cortes and Vapnik^28^ developed the SVM algorithm. This method is used for the LIB-SVM package to implement the SVM model for the MAP study^26,29^. Besides, SVM is based on linear regression with high dimensional attribute space using Cost (C), regression loss epsilon (ɛ), complexity bound (v), kernel functions, and numerical tolerance for parameter optimization. The main challenge in SVM is to select the Kernel function and best hyperparameter values. The^6,20,28,29^ papers discussed mathematical equations especially in the context of high-dimensional data and kernel functions. The mathematical equation for the two-dimensional dataset hyperplane is given by Eqs. (2) and (3):

2 $${x}_{2}=a{x}_{1}+b$$

3 $$ax_{1} - x_{2} + b = 0$$

Now, inputs $x=\left({x}_{1},{x}_{2}\right)$, a and b are the constants, and $w=\left(a,-1\right)$. The final linear equation is the hyperplane Eq. (4) as given below:

4 $$wx + b = 0$$

The further needs to find the function of the hyperplane, which may be useful to optimize deviations of the training dataset through Eq. (5):

5 $$f\left(x\right)=\sum_{i=1}^{l}{w}_{i} K\left({x}_{i}, x\right)+b$$

where $x$_i_ is the multi-dimensional input vectors, $K$ is a kernel function, and $l$ indicates the all-training data samples. This paper has obtained better prediction results using linear and polynomial Kernel functions^20,28,29^.

#### Decision tree

Decision Trees (DT) are easy to understand and give a better visual understanding. DT can take the non-linear relationships and interactions between different attributes. Meanwhile, DT helps to find the decision rules by using historical QS and MC datasets, which can provide significant factors influencing household electricity consumption. The next step is to use an ensemble-based Random Forest (RF) algorithm^27,30^.

A DT algorithm with a forward pruning approach is applied to get in this work. This algorithm handles categorical, numerical datasets and related problems. Gini Index (Gini Impurity) and Information Gain (Entropy) parameters are needed to identify the significant attributes. The parent and leaf nodes are assigned based on the entropy and Gini Impurity values. This process of splitting attributes into nodes can be used further for significant attribute selection. Equations (6–8) provides a mathematical representation of Gini, Information Gain, and Entropy respectively.

6 $$Gini=1-\sum_{i=1}^{C}{\left({p}_{i}\right)}^{2}$$

7 $$Information\, Gain=Entropy\, before\, splitting-Entropy\, after\, splitting$$

8 $$Entropy=\sum_{i=1}^{C}-{p}_{i}*{{\text{log}}}_{2}\left({p}_{i}\right)$$

where ${p}_{i}$ denotes the probability of a class, $C$ represents the number of classes. Information gain is applied to split attributes. The Gini Impurity and Entropy range ranges from 0 to 0.5 and 0 to 1, respectively. Thus, in terms of computing power, the Gini impurity is more efficient than the entropy parameter. The DT can reduce the estimate’s variance by increasing the number of trees, further increasing time and design complexity. This problem is overcome in the RF algorithm^26,30^.

#### Random forest

Random Forest (RF) is an ensemble method that mixes numerous DTs to improve predictive accuracy and reduce overfitting. Due to the dynamic and multiple factor dependency, the HEC prediction accuracy is low, and sometimes the overfitting issue is raised. In that case, RF is the one of the choice to overcome this problem. This is possible due to the RF’s ability to handle complex relationships of attributes and having multiple individual decision trees^12,15,23^.

The RF is based on a bagging technique as an ensemble approach using an individual set of decision trees. Moreover, RF can solve classification, regression, and other tasks. The RF was introduced by Tin Kam Ho and further developed by Leo Breiman and Adele Cutler^26^. The RF will not face an overfitting problem even though the number of trees may increase. Ideally, the limit depth of an individual tree (d) should be less than 10^4^. On the other hand, we have the highest prediction accuracy for the values of the hyperparameters, like N = 43 and d = 9. Moreover, each tree can be developed by randomly choosing the subset of variables, and the best variable is considered for tree split. The RF model includes averaging all individual tree performances as shown in Eq. (9).

9 $$\mathrm{RF\, classifiers\, is\, handleling\, tree\, based\, structure}=\mathrm{ h }\left({\text{x}},{\text{k}}\right)$$

where x is the input and individual distributed random vectors. Also $$,\mathrm{ k is the x input attribute used}$$ to collect the vote for each popular class^31^. The possibility of inputs ($$x)$$ attributes belonging to class $$c$$ is denoted by probability $$P \left[\left.c\right|{v}_{j}\left(x\right)\right]$$ represented in Eq. (10):

10 $$P \left[\left.c\right|{v}_{j}\left(x\right)\right]=\frac{P \left[\left.c\right|{v}_{j}\left(x\right)\right]}{\sum_{k=1}^{{n}_{c}}P \left[{c}_{k},{v}_{j}\left(x\right)\right]}$$

whereas class c $=1, 2, \dots ., {n}_{c}$. After splitting all trees, the value of $P \left[\left.c\right|{v}_{j}\left(x\right)\right]$ is approximately equal to one. This ${v}_{j}\left(x\right)$ is the training set for assigning inputs into class. When the discriminant function ${g}_{c}\left(x\right)$ is maximum, then only inputs $x$ are classified to class $c$.The mathematical representation in Eq. (11):

11 $${g}_{c}\left(x\right)=\frac{1}{{n}_{t}}\sum_{j=1}^{{n}_{t}}P \left[\left.c\right|{v}_{j}\left(x\right)\right]$$

#### Stochastic gradient descent

Gradient descent (GD) is a generic optimization algorithm that can be used in the optimization problem. For the loss function optimization, the hyperparameter learning rate is important; that can determine the step size in each iteration. SGD is one type of GD in which only one sample is randomly selected and performed each iteration. SGD is competent for large datasets and can handle online learning. It is suitable for situations where quick updates to the model are necessary. As the future monthly data can be updated, it may increase the dataset and also need the ability of online learning to help for better HEC prediction accuracy. Thus, SGD can help to get the prediction model dynamically, adapting to changing patterns as well^12,15,18,24^. The advantage of SGD is that it takes less time to train the model. The mathematical representation for SGD is given by Eqs. (12)–(14)^26,32^.

12 $${w}_{t+1}={w}_{t}-{\gamma }_{t}\nabla wQ\left({z}_{t},{w}_{t}\right)$$

13 $${w}_{t+1}={w}_{t}-{\gamma }_{t}g$$

14 $$g=\nabla wQ\left({z}_{t},{w}_{t}\right)$$

where is gradient, ${\{w}_{t},\mathrm{ t}=\mathrm{1,2},\dots ., n\}$ is the weight optimization stochastic process for a single random sample and ${z}_{t}$ $Q\left({z}_{t},{w}_{t}\right)$ is the loss function.

#### Multi-layer perceptron

**Figure 5:**
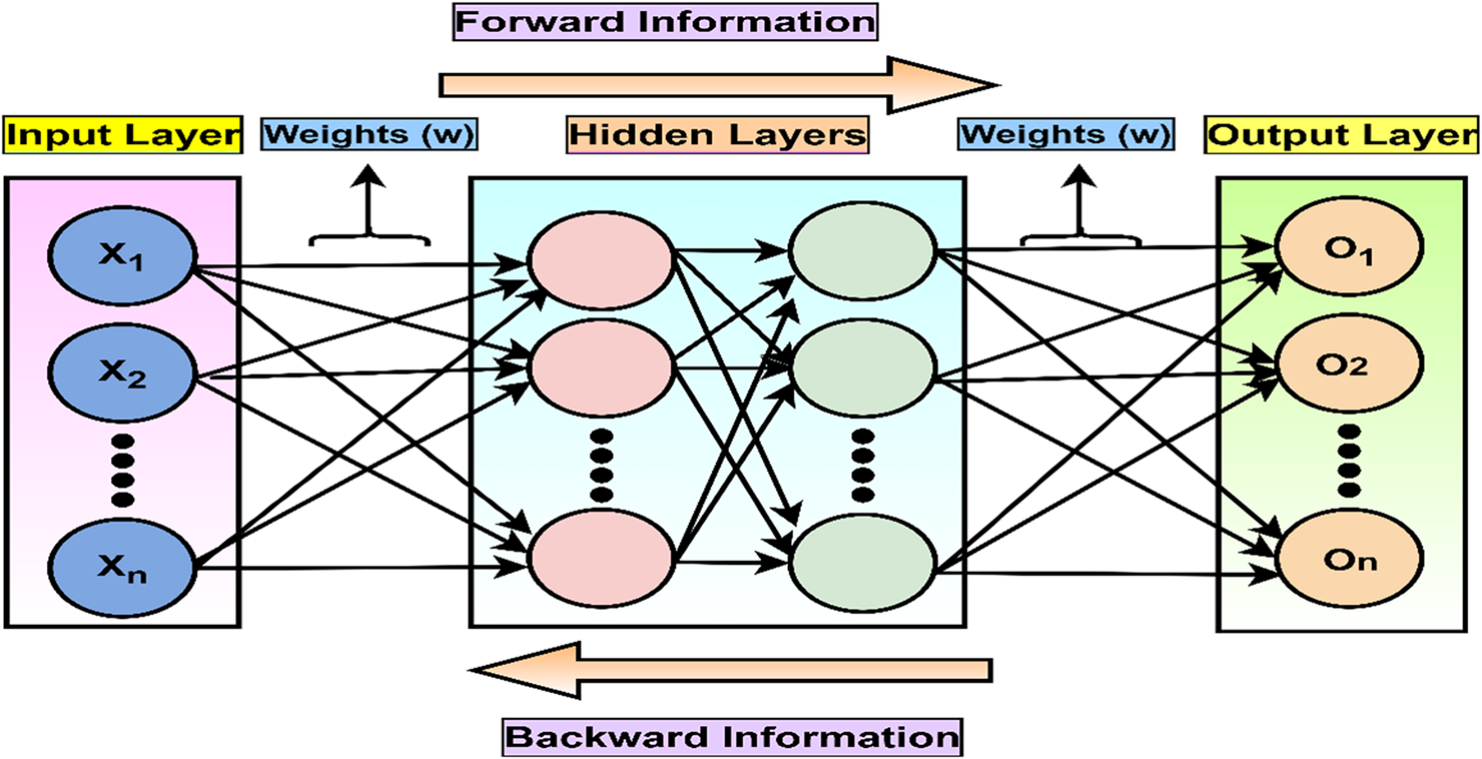
Architecture of artificial neural network.

Multi-layer Perceptron (MLP) is a type of NN capable of learning both complex, non-linear relationships in the attributes. The HEC prediction problem belongs to both complex and non-linearity types. The specialty of MLPs is to handle the complicated dependencies in HEC attributes, particularly when there are non-linear and shared effects between various attributes in the datasets^9,12,23^. The architecture of MLP is of ANN, which consists of three main layers, namely input, hidden, and output, as shown in Fig. 5^33^.

The only hidden layer performs the computational task on attributes compared to the input and output layers. Besides, we have implemented the MLP algorithm with the backpropagation technique. This Sklearn MLP algorithm is used to learn both linear and non-linear models. Let $X=\left({x}_{1},{x}_{2}, \dots \dots {x}_{n}\right)$ are the input feature vectors. The formula calculates the overall input to output ${y}_{ni-j}$:

15 $${y}_{ni-j}={b}_{j}+\sum_{i=1}^{n}\left({x}_{i},{w}_{ij}\right)$$

where ${w}_{ij}$ is the weights in the form of a matrix and ${b}_{j}$ is the bias parameter. Further, Eq. (15) is the activation function input and output layer.

The hyperparameter fine-tuning method noted that the Identity (not operating activation) activation function provided overfitted prediction results. Thus, further fine-tuning with the widely used hyperparameter Rectifier Linear Unit (ReLu) activation function and Adam weight solver optimizer have provided better prediction results. In addition, active function is used to fire neurons and handle the complex tasks of non-linearity problems. The weights, bias or threshold values, and activation function are essential components of NN. In the case of backpropagation NN, each neuron’s weights and biases would be updated based on the prediction error at the output layer^26,34^.

#### Adaptive boosting

Adaptive Boosting (AB) is based on an ensemble method deliberately used to improve prediction model performance. The specialty of AB is that it focuses on the weaknesses of individual models by assigning more weight to misclassified instances to balance the attributes. In the HEC prediction, some attributes may have more influence due to their higher magnitude than others. This is an unbalanced issue that may add bias to the prediction model. AB can nullify this problem to help address imbalances and improve overall prediction accuracy^3,9,12,23^.

The specialty of the AB ensemble algorithm is that it helps to reduce bias and variance, thereby enhancing the model’s prediction accuracy. This work uses the DT algorithm as a weak learner along with AB. In addition, the AB algorithm was introduced by Yoav Freund and Robert Schapire, for which they achieved the Gödel Prize in 2003. Initially the working of AB modeling needs to define the weights ${w}_{j}$ in Eq. (16):

16 $${w}_{j}=\frac{1}{n}, \quad where, j=1, 2,\dots \dots ,n$$

Then, for each iteration and input to set training dataset for a weak learner ${Wl}_{i}\left(x\right)$ through assigned weights and calculate weighted error ${Err}_{i}$ as Eq. (17)

17 $${Err}_{i}=\frac{\sum_{j=1}^{n}{w}_{j}I\left({t}_{j}\ne {wl}_{i}\left(x\right)\right)}{\sum_{j=1}^{n}{w}_{j}}, I\left(x\right)=\left\{\begin{array}{c}0 \quad if x=false\\ 1 \quad if x=true\end{array}\right.$$

Furthermore, based on the ${Err}_{i}$ errors, weights are adjusted for input attributes ${\beta }_{i}$ is shown in Eq. (18);

18 $${\beta }_{i}={\text{log}}\left(\frac{\left(1-{Err}_{i}\right)}{{Err}_{i}}\right)$$

Continuously, to reduce the loss function by providing error value back to the previous stage to modify weights for each $i to N$ where $N$ is the number of weak learners. Finally, the output stage obtained the best prediction results, adjusting weak learner for dataset test $\left(x\right)$^26,35^.

### Heterogeneous ensemble prediction models for MAP

The aim of proposed heterogeneous ensemble model is to boost prediction accuracy of individual model, robustness, and generalization. Further to identify the influencing datasets and perfect fit algorithm and model. The prediction modeling is called a heterogeneous ensemble model because the total dataset is distributed in different dataset combinations and provided to the same learners^5^.

**Figure 6:**
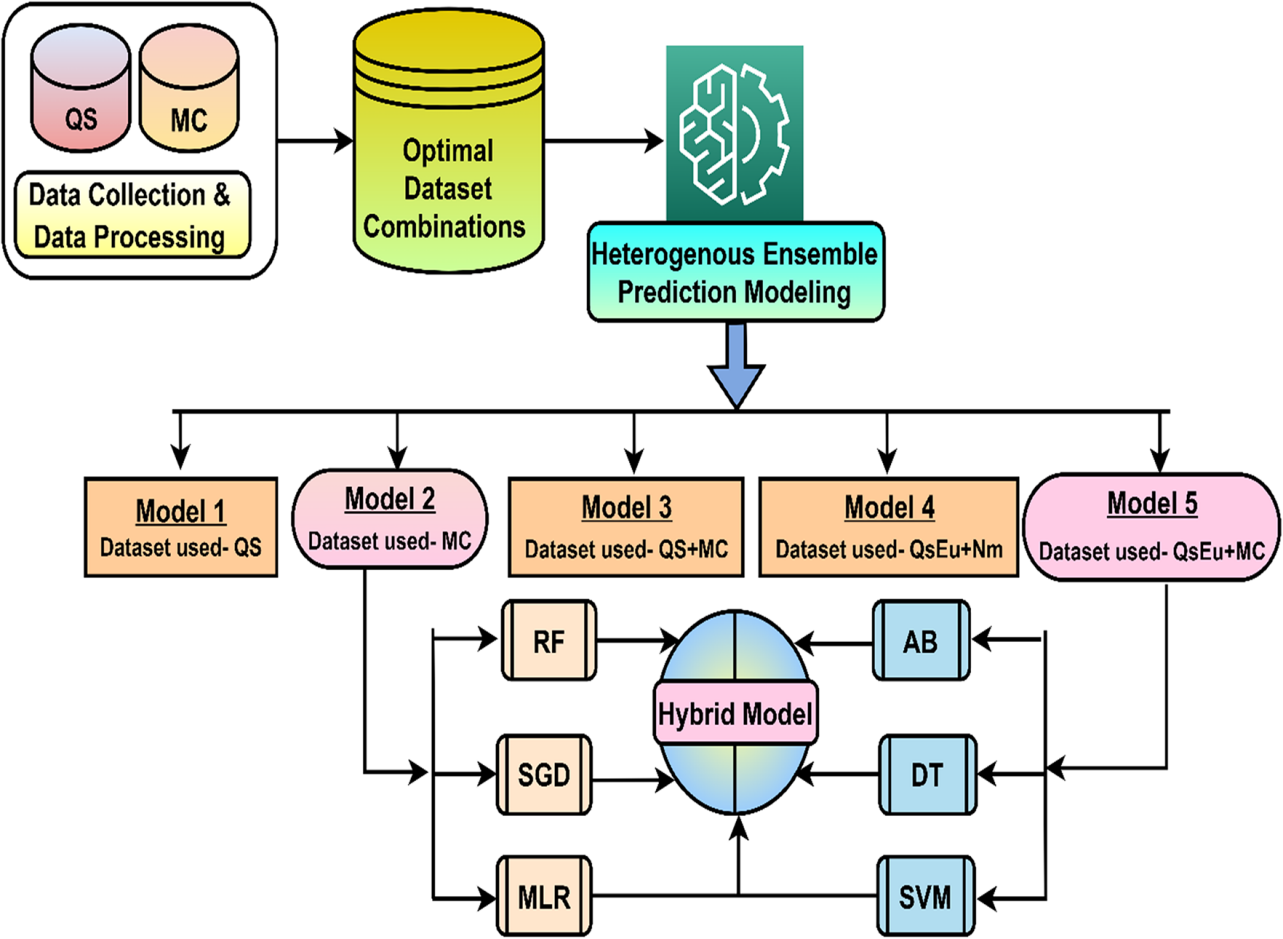
The proposed hybrid HEC prediction workflow.

Utilizing the ensemble’s diversity makes it feasible to identify more diverse patterns in the data, which enhances performance overall. The prediction model accuracy mainly depends on the availability of quality datasets, sufficient datasets, and an appropriate selection of algorithms and hyperparameters. This work is based on two original datasets, namely QS and MC. Further, QS and MC datasets are used to generate three more datasets using the data combination approach that is QS + MC, QS equation (QsEq) + next month’s consumptions (QsEqu + Nm), and QsEq + MC, as shown in Figs. 2 and 6. Thus, the prediction models used five datasets to form five heterogeneous ensemble models, as shown in Fig. 1. Model 1 to Model 5 are formed based on datasets like QS, MC, QS + MC, QsEq + Nm, and QsEq + MC, respectively. This five data-based model has been further used to develop month ahead prediction models as shown in Fig. 6.

### Proposed hybrid prediction model for MAP

The aim of proposed hybrid prediction model is to further increase prediction accuracy above 76%. The comparative study of five heterogeneous ensemble models concluded that model 2 (MC dataset) and model 5 (QsEqu + MC datasets) had less prediction error than the remaining dataset-based MAP models. Further, among each model top 3 best fitted algorithms are selected like Model 2 included RF (R), SGD (S) and MLR (M) and Model included AB (A), DT (D) and SVM (S) to form the hybrid month-ahead prediction model called SMSDAR as shown in Fig. 6. This approach of selection of models and algorithms helps to improve the prediction model performance by considering the diversity of features, datasets and algorithm’s strengths. Thus, the perfect fit prediction models specifically the best fitted algorithms output are used to develop the hybrid model. Table 1 shows the fine-tuned hyperparameters for Model 2 and Model 5 with the best fitted algorithms^12^, and Python programming and Orange data mining tool (version 3.31.0) were used to implement the prediction models^26^. During the prediction model development hyperparameters of all the algorithms fine-tuned to fit the input data for better prediction model performance.

**Table 1:** The hyperparameters of hybrid model of MAP.

Model	ML Algorithm	Hyperparameter	
2	RF	Number of trees	43
		Number of attributes considered at each split	45
		Maximum depth of the trees	9
		Minimum samples slits	2
	SGD	Regularization	Lasso (L1)
		Learning rate	Inverse scaling
		Initial learning rate (^η^_0_)	0.0004
		Number of iterations	1000
	MLR	Regularization technique	Lasso (L_1_)
		Regularization strength (α)	300
5	AB	Base learner (weak learner)	DT
		Number of learners	115
		Learning rate	0.999
		Regression loss function	Linear
	DT	Induce binary tree	True
		Minimum number of iterations in leaves	1
		Stop when majority reaches (%)	100
	SVM	Regression Cost (C)	0.60
		Kernel function	Linear
		Numerical tolerance	0.0001
		Iteration limit	100

Passos and Mishra^36^ have provided insights on how to use hyperparameters. This study applied three evaluation parameters for prediction model performance analysis: RMSE, MAE, and R^2^. A mathematical representation of evaluation parameters is given in Eqs. (19)–(21)^35^.

19 $$RMSE=\sqrt{\frac{\sum_{i=1}^{n}{(\left|{y}_{i}-{\widehat{y}}_{i}\right|)}^{2}}{n}}$$

20 $$MAE=\frac{\sum_{i=1}^{n}\left|{y}_{i}-{\widehat{y}}_{i}\right|}{n}$$

21 $${R}^{2}=1-\frac{\sum_{i=1}^{n}{(\left|{y}_{i}-{\widehat{y}}_{i}\right|)}^{2}}{\sum_{i=1}^{n}{(\left|{y}_{i}-{\widehat{y}}_{i}\right|)}^{2}}$$

where $n$ is the number of instances as houses, ${y}_{i}$ is the i^th^ input house, and ${\widehat{y}}_{i}$ is the corresponding prediction output.

## Results and discussion

The nature of the HEC pattern is dynamic and complex. The reason is its dependency on various factors like socio-demographics, usage of appliances, lifestyle, weather conditioning, etc. Thus, obtaining accurate HEC prediction is a challenging task. This work has addressed the prediction accuracy challenge and improve the accuracy by proposing a novel methodology. However, the prediction models are developed using the ten-fold cross-validation technique. Ten-fold cross-validation is applied because it balances computational efficacy with offering an accurate assessment of the model. Whereas, the proposed prediction model works with a robust validation approach through tenfold cross-validation to address potential limitations or uncertainties in the original results. This section discusses the implementation of methods and results obtained at each stage in a stepwise manner.

### Dataset quality improvement and its assessment using a three-stage approach

A three-stage approach is implemented to improve the quality of original datasets QS and MC. Conversely, this step helps improve the quality of the other three generated datasets. The data QA has a direct influence on increasing HEC prediction accuracy. The three-stage process of data QA is applicable for QS and MC separately, as shown in Fig. 3. For the QS dataset, stages are before, after, and after correlation with feature engineering. The prediction model performance and complexity is depending on the selection of optimal and significant features or attributes. Figure 4, Tables 2 and 3 shows that how the selection of significant attributes affecting on the prediction model performance. Thus, based on the type of attributes Pearson and Spearman correlation statistical methods is used to identify the significant features on energy consumption from 65 attributes.

**Table 2:** Data quality assessment for the QS dataset.

Models	Stage I: before correlation			Stage II: after correlation			Stage III: after correlation & FE		
	RMSE	MAE	R^2^	RMSE	MAE	R^2^	RMSE	MAE	R^2^
MLR	69.28	53.00	0.13	68.99	52.03	0.14	67.24	51.47	0.18
SVM	66.58	51.60	0.20	66.65	51.71	0.20	66.13	51.23	0.21
DT	73.48	56.49	0.024	72.35	55.96	0.05	72.66	55.18	0.05
RF	67.89	52.59	0.17	66.61	51.11	0.20	66.71	51.11	0.19
SGD	66.34	50.94	0.20	65.87	49.69	0.22	66.43	51.25	0.20
MLP	72.42	54.13	0.05	70.44	53.17	0.10	69.67	52.70	0.12
AB	67.90	51.94	0.17	69.02	50.32	0.14	67.30	50.29	0.18

RMSE and MAE in kWh.

**Table 3:** Data quality assessment for the MC dataset.

Models	Stage I: before preprocess			Stage II: after preprocess			Stage III: after preprocess & FE		
	RMSE	MAE	R^2^	RMSE	MAE	R^2^	RMSE	MAE	R^2^
MLR	44.64	31.96	0.64	39.22	28.00	0.72	39.51	28.23	0.72
SVM	41.86	30.51	0.68	41.37	30.59	0.69	40.77	31.83	0.70
DT	45.20	30.89	0.63	45.81	31.41	0.62	39.89	27.11	0.71
RF	39.89	27.37	0.71	37.56	26.51	0.74	36.18	25.73	0.76
SGD	42.16	31.10	0.68	41.92	30.27	0.68	39.51	28.36	0.72
MLP	56.16	37.50	0.43	46.27	32.19	0.61	50.93	36.4	0.53
AB	39.85	27.22	0.71	37.26	26.80	0.75	36.72	26.37	0.76

RMSE and MAE in kWh.

The feature engineering is the one of the data quality improvement technique under data preprocessing. Based on understanding the data, problem statement and creating new features from the existing features is the FE technique. This technique is used to add new 5 features in QS and MC dataset. Further, the newly added features also contributed in the prediction model performance.

In addition, except for SVM, the remaining ML algorithms have increased prediction accuracy. The best prediction accuracy is provided by SGD to RMSE (65.87 kWh), MAE (49.69 kWh), and R^2^ (0.22), as shown in Table 2. Still, the prediction performance for QS before and preprocessing is poor. Further, the FE technique has been used to increase prediction accuracy. The FE-based generates five features: the sum of socio-demographic parameters, total regular appliances, total lifestyle appliances, number of five-year-old appliances, and QsEq. The sum of socio-demographic parameters includes selected attributes like districts, rooms, windows, and members. Regular appliances include a TV, ceiling fan, mobiles, mixer, water purifier, and CFL bulbs. The lifestyle appliances include AC, exhaust fan, electric geyser, zero bulbs, desktop, dongle, Wi-Fi, home audio system, and electronic games. The next feature is how many appliances are more than five years old in the house. The QsEq is the summation of all the remaining attributes.

In addition, except for DT, SGD, and RF, the remaining ML algorithms have increased prediction accuracy. The best prediction accuracy is provided by SVM to RMSE (66.13 kWh), MAE (51.23 kWh), and R^2^ (0.21), as shown in Table 2.

Similarly, the MC dataset followed steps before, after preprocessing, and after preprocessing with FE, as shown in Fig. 3. The MC datasets observed issues like irregular energy consumption of 49 houses and average billings of 43 homes that have been corrected. These issues are resolved by removing irregular consumption houses and applying the average window method to correct average bills. In addition, except for DT and SGD, the remaining ML algorithms have increased prediction accuracy. The best prediction accuracy is achieved for AB to RMSE (37.26 kWh), MAE (26.80 kWh), and R^2^ (0.75), as shown in Table 3. It is also observed that MLR ML algorithms have increased prediction accuracy faster than other ML algorithms, from R^2^ = 0.64 to R^2^ = 0.72.

Furthermore, the FE approach is used to increase the prediction accuracy. The FE has added five attributes to the MC dataset: actual minimum consumption (Considered above 3 kWh per month), maximum consumption, standard deviation, actual monthly summation (considered above 3 kWh and normal monthly consumption), and actual average consumption.

All the ML algorithms that has been considered have increased prediction accuracy except MLP. The best prediction accuracy is achieved for RF to RMSE (36.18 kWh), MAE (25.73 kWh), and R^2^ (0.76), as shown in Table 3. Initially, MLR and MLP provided overfitted prediction results for the MC dataset. This issue is talked about by selecting appropriate hyperparameters. After this, MLR has increased the prediction accuracy and is considered in the hybrid model development. Thus, Tables 2 and 3 show the increased prediction accuracy of the MAP model from the first stage to the third stage. Hence, the third stage-based QS and MC dataset achieved data quality and identified the perfect fit ML algorithms, as shown in Tables 2 and 3.

### Heterogeneous ensemble MAP model analysis

**Figure 7:**
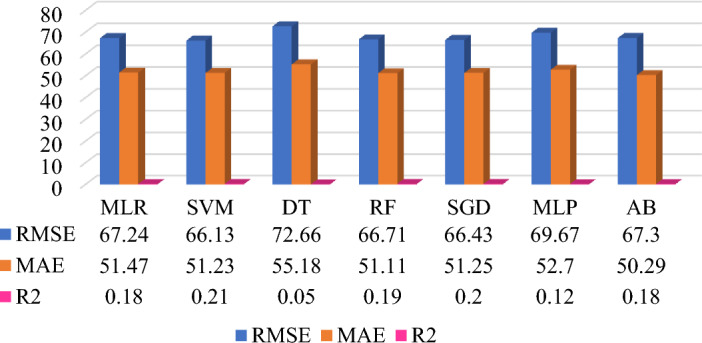
QS dataset-based MAP model (energy consumption in kWh).

**Figure 8:**
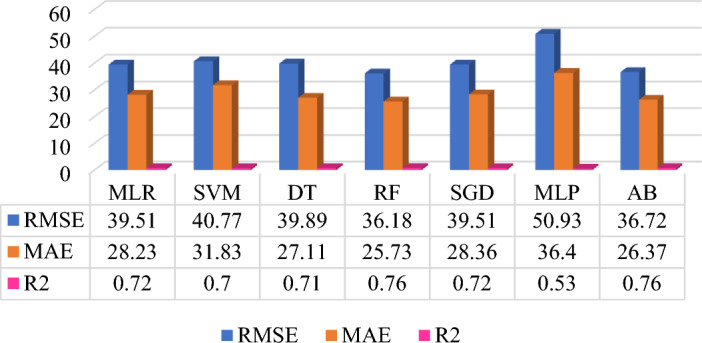
MC dataset-based MAP model (energy consumption in kWh).

**Figure 9:**
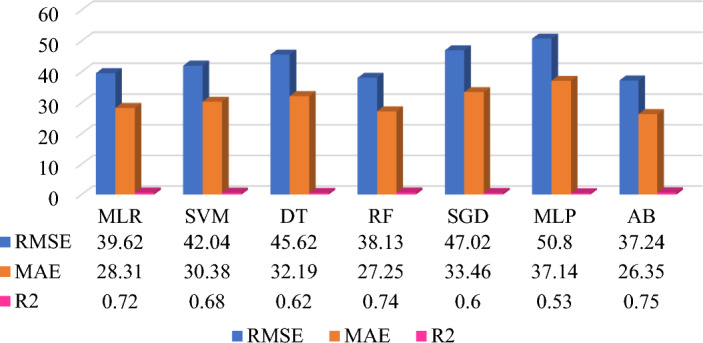
QS + MC dataset-based MAP model (energy consumption in kWh).

**Figure 10:**
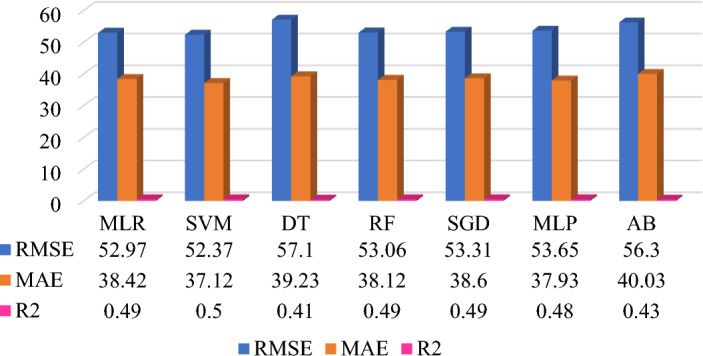
QsEq + Nm dataset-based MAP model (energy consumption in kWh).

**Figure 11:**
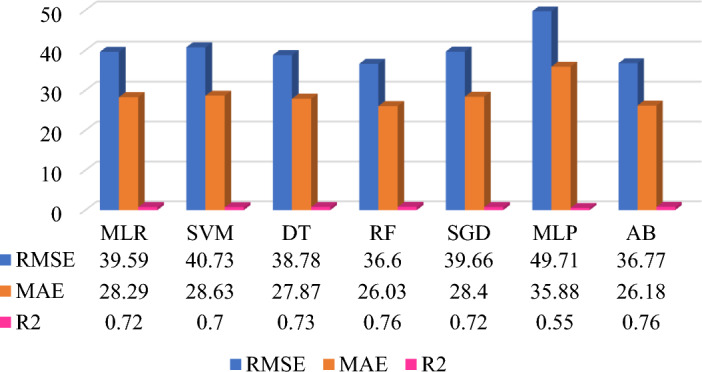
QsEq + MC dataset-based MAP model (energy consumption in kWh).

The prediction model performance can be increased by selecting the significant features, data quality improvement in preprocessing, type of datasets, selection of ML algorithms, and fine-tuning of the hyperparameters. Five different datasets are applied on same seven base learner or algorithms to form the heterogeneous ensemble method. The prediction model development is based on supervised machine learning technique which includes the average monthly energy consumption (Avg_kWh) feature is target feature for all five prediction models. Each dataset has included different features which influences on the prediction model performance. Figures 7, 8, 9, 10, and 11 shows the prediction model performance based on dataset. The model performance is analyze using evaluation matrix namely RMSE, MAE and R^2^.

Prediction Model 1 is developed using QS data and seven ML algorithms. Figure 7 shows the comparative analysis of individual prediction model with the evaluation matrix. It indicates that SVM is providing the best prediction performance to RMSE (66.13 kWh), MAE (51.23 kWh), and R^2^ (0.21) followed by Random Forest model. The highest prediction model accuracy is around 21% of underfitting for QS datasets. The prediction model 1 also shows that the Decision Tree and Stochastics Gradient Descent algorithms are unfit to QS data. Whereas, the ensemble models like RF and AB work are more preferable to deal with QS data. The inference of the prediction accuracy is the, QS data includes features related to household characteristics, appliances, and socio-demographic information, and the target feature is monthly energy consumption.

The QS data includes linear, non-linear, and multiple-magnitude features. Thus only the QS data is not sufficient to get boosted prediction model performance. But the features used in QS data are essential to understand household energy consumption pattern. Prediction Model 2 is developed using MC data and applied the same algorithms that are used in Model 1, as shown in Fig. 8. The comparative result analysis of Model 2 show that highest prediction accuracy is 76%, achieved by Random Forest and Adaptive Boosting algorithms and followed by MLR and SGD. All algorithms are fitting to MC data except MLP to give average around 72% of prediction accuracy. As compared to Model 1, Model 2 has drastically increased the prediction accuracy which indicates the MC data is fitting better than QS data. The inference of better prediction performance is the input and output features are uniformed and of same type. Thus, for boosting the prediction accuracy, MC data can be used for developing monthly energy consumption prediction model. But the MC data missed other significant attributes that are covered in QS data. Thus the combination of QS and MC datasets can be the suitable choice to develop robust model.

Further, existing QS and MC datasets are integrated using common household ID feature to generate new QS + MC dataset. The new data is generated data by Features Engineering technique to check the prediction model performance. This is one attempt to check the significance of new data and features on prediction model accuracy. Thus, prediction Model 3 uses QS + MC dataset and the same algorithms used in Model 1 and Model2. Figure 9 shows the comparative prediction model performance using different algorithms. Among this, AB provides the best prediction performance to RMSE (37.24 kWh), MAE (26.35 kWh), and R^2^ (0.75) followed by RF and MLR. The prediction results show that the QS + MC dataset is fitted to ensemble algorithms like AB and R and MLP is still underfit compared to other algorithms, as shown in Figs. 8 and 9. Moreover, performance of the prediction Model 3 is slightly decreased compared to Model 2 but still better than that of Model 1. Thus, the combination of QS and MC data is good choice for household energy consumption prediction.

The next attempt is to improve the prediction model performance by considering selected features from QS and MC datasets. The optimal feature can help to reduce the model complexity, increase the computation speed and prediction model performance. Prediction Model 4 uses one feature from QS which is obtained by arithmetic sum (QsEq) of the significant features from QS data and another features from MC data that is considering only previous years’ monthly energy consumption (Nm) of the target month. This features and data are provided to the same algorithms and fine-tuned hyperparameters to develop prediction model 4. Figure 10 shows the comparative prediction Model 4. The result indicates that SVM provides the best prediction performance to RMSE (52.37 kWh), MAE (37.12 kWh), and R^2^ (0.50) as compared to other algorithms. It is also observed that due to balancing the attributes from QS and MC datasets, all the ML algorithms provided the highest prediction accuracy, around 50%. Model 4 is uses least features as compared to remaining models. Whereas, Model 4 achieved improved prediction performance compared to Model 1 but less than Models 2 and 3. Thus, QsEq feature is significant feature of QS data and which is generated by feature engineering technique.

The last attempt for improving the prediction model performance is to combine most significant features and data namely QsEq and MC dataset to form Model 5. The QsEq + MC data is provided to same algorithms used in previous model and fine-tuned hyperparameters to get better prediction performance. Figure 11 shows the comparative prediction model analysis for Model 5. The prediction result shows that RF provides the highest 76% prediction accuracy with RMSE (36.60 kWh), MAE (26.03 kWh), and R^2^ (0.76) followed by AB to RMSE (36.77 kWh), MAE (26.18 kWh), and R^2^ (0.76). It is observed that for Model 2 the same performance is obtained by RF and AB algorithms. This indicates that the RF and AB ensemble algorithms fits the QsEq + MC data, in that MC data includes more features compared to QS data. However, the MLP algorithm is not fitting to all data combinations for this prediction problem. Overall the prediction models are handled different model complexity and non-linearity to get the prediction model performance.

### Proposed SMSDAR hybrid MAP model to increase prediction accuracy

The seven heterogeneous ensemble prediction model are developed and achieved up to 76% prediction accuracy by Model 5 as shown in Fig. 11. There are different methods to developed prediction model like Average, token, stacking etc., to improve the prediction accuracy of the individual models^3,6^. The selection of this model dependences on the type of problem, data and required outcome. Here, the tolerance based approach is used to develop the hybrid model. In this approach based on tolerance of predicted result is calculated and the final expected output is selected based on less tolerance^37^. This way each model’s algorithms are contributing to boost the final prediction model performance called SMSDAR hybrid model. The SMSDAR is formed using combination of algorithms mentioned here, SVM + MLR + SGD + DT + AB + RF as shown in Fig. 6.

Thus, input of hybrid model is output of heterogeneous ensemble individual model and further need to explored prediction results to analyses prediction tolerance and calculate the prediction performance. The highest prediction accuracy with less tolerance of each ML algorithm is considered as the predicted output. The proposed SMSDAR hybrid model has increased prediction accuracy from 76 to 92% as shown in Table 4. The results of the proposed hybrid model show the increased prediction accuracy of RMSE (22.02 kWh), MAE (13.04 kWh), and R^2^ (0.92).

**Table 4:** Comparative study of individual models with hybrid model.

Sr. No	Model No	Dataset	RMSE	MAE	R^2^
1	Model 5	QsEq + MC	40.73	28.63	0.70
2	Model 2	MC	39.51	28.23	0.72
3	Model 2	MC	39.51	28.36	0.72
4	Model 5	QsEq + MC	38.78	27.87	0.73
5	Model 5	QsEq + MC	36.77	26.18	0.76
6	Model 2	MC	36.18	25.73	0.76
7	SMSDAR Model	QS & MC	22.02	13.04	0.92

### Threats to validity for prediction model results

A direct prediction model performance comparison of proposed results and the existing models results is challenging. This is due to the proposed prediction models are developed on real world problem statement on first hand collected data with specific features. However, there is variations in the implications approach, applied proposed methodology, used data-collection methods, selected datasets, data features and algorithms. The assessment, validation indicators that can be applied differently in different works, which can vary the results of prediction models. The performance of the prediction model can be compared or verified when the model works on the similar data, methodology, and implications. In comparison, every prediction ML algorithm has its potential and limitations. Thus, one algorithm cannot perform the best on all data sets and applications under all conditions^12,38^. Whereas, the energy consumption prediction of individual household electricity is dynamic and depends on various direct and indirect parameters like life style, geographical locations, household characteristic, weather conditioning etc. However, the proposed study is region and couldn’t get sufficient exiting prediction results as benchmark to compare. Due to these threats, the proposed prediction model results cannot have compared with the existing techniques or models for the household energy consumption study.

## Conclusion and future scope

Electrical utilities face the challenge of fulfilling the increased demand for electricity. For this, utilities should know consumers’ electricity consumption patterns and accurate electricity consumption predictions. Meanwhile, HEC is dynamic, and consumption depends on multiple factors, which limits prediction accuracy. This problem is addressed through a proposed novel methodology to boost the prediction accuracy of residential consumers. The information from 225 houses’ QS and MC datasets is applied to the developed MAP model. The accuracy of the MAP model is boosted by using approaches like correlation methods, FE techniques, data quality assessment, heterogeneous ensemble prediction (HEP), and the novel hybrid model. Spearman and Pearson correlation methods were used to optimize attributes of the QS dataset. The MC dataset has two challenges, that is, irregular energy consumption, and average billing problems.

Thus, irregular consumption houses were removed and considered the corrected average billing houses using the average window method. The preprocessed QS and MC datasets are used to generate three more datasets, namely QS + MC, QS equation (QsEq) + next month’s consumptions, and QsEq + MC. The five HEP models were developed using five datasets and seven ML algorithms were used for prediction. After fine-tuning hyperparameters with ten-fold cross-validation method, MC (Model 2) and QsEq + MC (Model 5) dataset-based models provided higher prediction performance. In Model 2, RF provided better performance with RMSE (36.18 kWh), MAE (25.73 kWh), and R^2^ (0.76). Similarly, in Model 5, AB is also provided better performance concerning RMSE (36.77 kWh), MAE (26.18 kWh), and R^2^ (0.76). The prediction accuracy is further increased using the proposed hybrid model. The new hybrid model consists of perfect fit ML algorithms, namely SVM (S), MLR (M), SGD (S), DT(D), AB (A), and RF (R) called the SMSDAR hybrid model. This hybrid model achieved a prediction accuracy of RMSE (22.02 kWh), MAE (13.04 kWh), and R^2^ (0.92). In contrast, the MLP and MLR algorithms faced overfitting problems which was resolved through hyperparameters tuning.

As a future scope of the proposed work, deep learning algorithms can be used to improve prediction accuracy. The proposed work is an initiative that can support the future smart grid development. For the effective implementation of this work in smart grid a priori work is needed on real-time demand response management in microgrid along with active participation of consumers. Also, for a cost effective design of conventional electricity grids, consumer participation is crucial. This can happen through providing a mobile app or webpage-based access for consumers. Moreover, the consumer participation helps to identify and resolve the consumer’s issues as well. This feedback-based system may enhance utility companies’ performance and support the nation’s smart grid development.

## Data Availability

The datasets generated and/or analysed during the current study are not publicly available due to the data that has been used being confidential, and the study is yet to be completed but is available from the corresponding author on reasonable request.
